# Viral Double-Strand RNA-Binding Proteins Can Enhance Innate Immune Signaling by Toll-Like Receptor 3

**DOI:** 10.1371/journal.pone.0025837

**Published:** 2011-10-10

**Authors:** Yvonne Lai, Guanghui Yi, Alice Chen, Kanchan Bhardwaj, Brady J. Tragesser, Rodrigo A. Valverde, Adam Zlotnick, Suchetana Mukhopadhyay, C. T. Ranjith-Kumar, C. Cheng Kao

**Affiliations:** 1 Department of Molecular & Cellular Biochemistry, Indiana University, Bloomington, Indiana, United States of America; 2 Department of Plant Pathology and Crop Physiology, Louisiana State University Agricultural Center, Baton Rouge, Louisiana, United States of America; 3 Biology Department, Indiana University, Bloomington, Indiana, United States of America; University of Texas Medical Branch, United States of America

## Abstract

Toll-like Receptor 3 (TLR3) detects double-stranded (ds) RNAs to activate innate immune responses. While poly(I:C) is an excellent agonist for TLR3 in several cell lines and in human peripheral blood mononuclear cells, viral dsRNAs tend to be poor agonists, leading to the hypothesis that additional factor(s) are likely required to allow TLR3 to respond to viral dsRNAs. TLR3 signaling was examined in a lung epithelial cell line by quantifying cytokine production and in human embryonic kidney cells by quantifying luciferase reporter levels. Recombinant 1b hepatitis C virus polymerase was found to enhance TLR3 signaling in the lung epithelial BEAS-2B cells when added to the media along with either poly(I:C) or viral dsRNAs. The polymerase from the genotype 2a JFH-1 HCV was a poor enhancer of TLR3 signaling until it was mutated to favor a conformation that could bind better to a partially duplexed RNA. The 1b polymerase also co-localizes with TLR3 in endosomes. RNA-binding capsid proteins (CPs) from two positive-strand RNA viruses and the hepadenavirus hepatitis B virus (HBV) were also potent enhancers of TLR3 signaling by poly(I:C) or viral dsRNAs. A truncated version of the HBV CP that lacked an arginine-rich RNA-binding domain was unable to enhance TLR3 signaling. These results demonstrate that several viral RNA-binding proteins can enhance the dsRNA-dependent innate immune response initiated by TLR3.

## Introduction

Viral nucleic acids are agonists for innate receptors that can activate anti-viral responses [1], [2], [3], [4]. The receptors include the RIG-I-like receptors that are localized to the cell cytoplasm and several membrane-associated Toll-like receptors [5], [6]. Mutations in these receptors have been correlated with pathologies in viral infections in humans [7], [8], [9], [10]. Understanding how the innate immune receptors can interact with agonists will be important for the detection and response to viral infections.

Toll-like Receptor 3 (TLR3), the focus of this study, is composed of a large ligand-binding ectodomain, a transmembrane helix, and a signaling Toll/IL-1 receptor homology (TIR) domain [11], [12]. The ectodomain contains 23 leucine-rich repeats flanked with cysteine-rich caps and a bipartite motif, both of which are critical for ligand binding [13], [14]. TLR3 localizes to either the plasma membrane or acidic endosomes, the latter was presumed to be the site of high affinity binding to dsRNA [15], [16]. Ligand binding induces the TLR3 dimer to orient the two TIR domains to recruit adaptor proteins and activate signal transduction [6], [14].

Karioko et al. [17] have observed the activation of TLR3 signaling in cultured cells with complex ligand mixtures, such as nucleic acids fractions from necrotic cells. However, we have anecdotally observed that highly purified RNAs, including necrotic RNAs, are poor activators of TLR3 in cultured cells when compared to the synthetic dsRNA analog, poly(I:C). These observations suggest that intrinsic features within dsRNAs can influence signaling by TLR3. Also, component(s) in addition to the dsRNAs are needed to activate TLR3. The antimicrobial peptide LL37 and other peptides that can bind dsRNA were recently found to enhance the TLR3 response to poly(I:C) and viral dsRNAs [18]. Since viral RNAs exist as complexes with proteins, we wanted to determine whether viral proteins could act to modulate TLR3 signaling.

In this work, we determined that viral dsRNA-binding proteins could substitute for LL37 in activating signaling by TLR3. Furthermore, the proteins promoted more efficient recognition of viral dsRNAs. The recombinant polymerases from the Hepatitis C virus (HCV) and three viral capsid proteins were characterized in this study.

HCV, a member of the *Hepacivirus* genus in the *Flaviviridae* family, infects approximately 2% of the world's population, with up to 80% of untreated individuals progressing to chronic infection and severe liver damage [19], [20]. The HCV-encoded polymerase, NS5B, is a validated drug target and has been extensively characterized [21]. As with polymerases in general, NS5B resembles a closed right hand that contains thumb, palm and finger subdomains [19]. *In vitro*, recombinant NS5B proteins can bind and initiate RNA synthesis by a *de novo* initiated mechanism or by extension from a template annealed to a primer [22], [23], [24], [25], [26], [27], [28]. *De novo* initiated RNA synthesis uses a single-stranded (ss) RNA template and a purine nucleotide as the first nucleotide, while primer extension uses a partially duplexed RNA [28]. The *de novo* initiated versus primer-extended modes of RNA synthesis is regulated by a flexible loop named Δ1 that extends from the fingers subdomain to contact hydrophobic residues in the thumb subdomain [27]. The release of the Δ1 loop is needed to sterically accommodate the ternary complex during elongative synthesis. The polymerase from the 2a genotype of HCV exists in a structurally more closed conformation than that of the 1b polymerase in part due to a tighter contact between the Δ1 loop and the thumb subdomain [27], [29], [30]. This feature has been proposed to allow the 2a polymerase to be more efficient at RNA synthesis by the *de novo* initiated mechanism since it cannot initially accommodate a partially duplexed RNA.

Two of the viral capsid proteins we examined are from members of the alphavirus-like superfamily with positive-stranded RNA genomes: Ross River virus (RRV) and Brome mosaic virus (BMV) [31], [32]. The third is from the orthohepadnavirus, Hepatitis B virus (HBV), which has a partially dsDNA genome and replicates by reverse transcription [33], [34]. The capsids of all three viruses contain a positively-charged arginine-rich region that is required for RNA binding [35], [36], [37], [38], [39].

## Results

### Viral dsRNAs are poor agonists for TLR3

**Figure 1:**
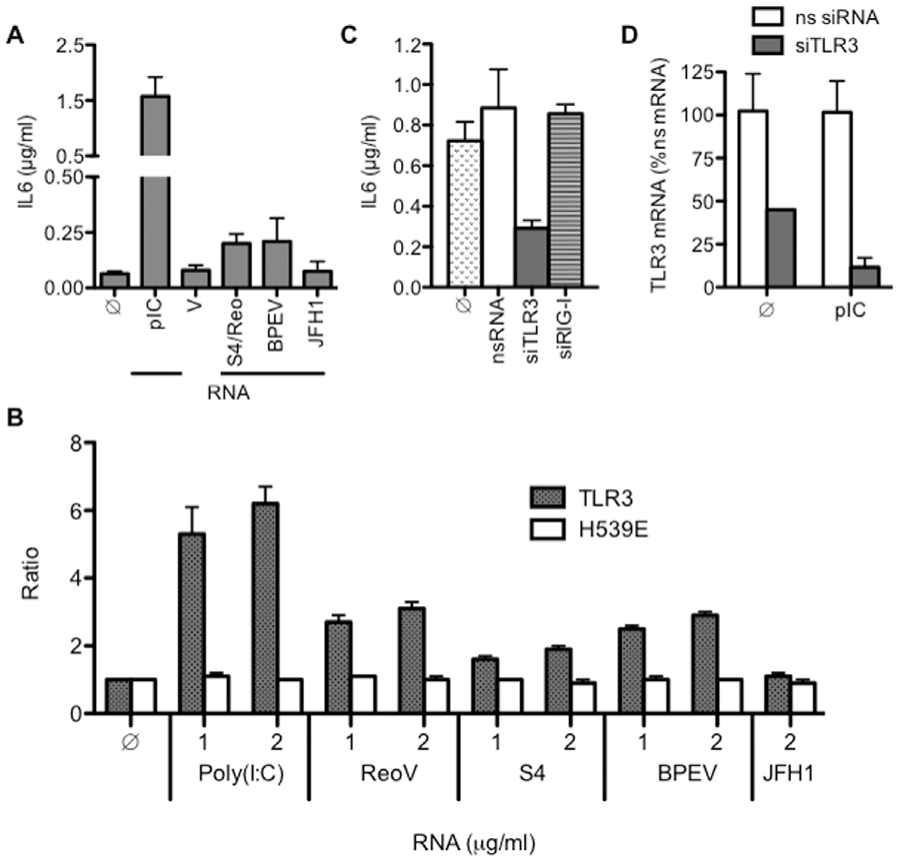
A comparison of viral dsRNAs and poly(I:C) for activation of TLR3. A) Effects of various potential TLR3 ligands on IL6 production by BEAS-2B cells. All of the RNAs were added to the culture media. Poly(I:C) (pIC) was added to 0.13 µg/ml and the other RNAs were added at 0.13 to 0.3 µg/ml. IL6 was quantified in an aliquot of clarified cell medium, and the total amount of IL6 was normalized to the total medium volume. The data shown is the mean ± SEM, with the number of assays in parentheses: pIC (n = 23), Reovirus dsRNA (ReoV, n = 9), Reovirus S4 dsRNA (S4; n = 28), BPEV (n = 6), JFH1 (n = 3). B) A comparison of the effects of viral dsRNAs and poly(I:C) in HEK 293T cells transfected to express the WT TLR3 or a ligand-binding mutant, H539E. Activation of TLR3 was measured by the ratio of the firefly luciferase driven by an ISRE promoter element to a constitutively-expressed *Renilla* luciferase [47]. Each bar represents the mean value of three replicates, with the range for one standard error shown above the bars. C) TLR3 is required for the increase in IL6 production in response to BPEV dsRNA. BEAS2B cells were transfected with a control nonspecific siRNA (nsRNA), a set of three siRNAs to TLR3 (siTLR3), or a siRNA to RIG-I (siRIG-I), grown for 48 h before addition of BPEV dsRNA (0.3 mg/ml) to the culture media. D) RT-PCR results demonstrating that the siRNA targeting TLR3 decreased the abundance of the TLR3 message. BEAS2B cells were transfected with either control nonspecific siRNA or siRNAs to TLR3. After 48 h, cells were exposed to media alone (f) or to poly(I:C) at 0.13 µg/ml. RT-PCR was performed to determine fold induction of TLR3 mRNA above media 18 h later. Data is presented as a percent of the result from the nonspecific siRNA.

We have anecdotally observed that cultured cell lines that express TLR3 respond poorly to viral dsRNAs when compared to the response to poly(I:C). To document this observation, BEAS-2B cells that endogenously express TLR3, TLR4, and RIG-I were examined for their response to several dsRNAs. BEAS-2B cells have been used extensively to study the effects of viral infection and activation of innate immunity on cytokine production, including the production of Interleukin 6 (IL6) [40], [41], [42]. The dsRNAs tested include the Reovirus genomic RNA, the S4 dsRNA made by annealing *in vitro* transcribed positive- and negative-sense strands of the Reovirus S4 RNA, and the Bell pepper endornavirus dsRNA, BPEV, purified from bell pepper plants [43], and other viral and synthetic dsRNAs. Transcripts of the JFH-1 genome were used as a ssRNA control. All the RNAs were added to the media of BEAS-2B cells and the amount of cytokine IL6 secreted into the medium quantified by ELISA (Fig. 1A). At 20 h, IL6 levels induced by poly(I:C) (0.13 µg/ml) was at least six-fold higher than that of dsRNAs or ssRNA (0.3 µg/ml; Fig. 1A and Fig. S1A). The production of IL6 in response to poly(I:C) was rapid and could be observed within 2 h of poly(I:C) addition (Fig. S1B). In contrast, cells treated with S4 dsRNA did not yield level of IL6 significantly above the background even after 20 h (Fig. S1B).

HEK 293T cells do not detectably express TLR3 but can be transfected to express either wild-type or mutant TLR3 [44]. Transfection of two plasmids, one containing an interferon stimulated response element (ISRE) promoter-driven firefly luciferase and a second encoding a constitutively expressed *Renilla* luciferase allow the analysis of TLR3 activation by different RNAs. HEK 293T cells expressing WT TLR3 responded to poly(I:C) (1–2 µg/ml), better than viral dsRNAs (1–2 µg/ml) purified from Reovirus and BPEV (Fig. 1B). Annealed S4 Reovirus dsRNA, ssRNAs from JFH-1 (Fig. 1B), endornavirus dsRNA from rice plants and other viral ssRNAs (Fig. S1C) also had no effect on TLR3 signaling. None of RNA tested induced reporter activity in HEK 293T cells expressing a TLR3 mutant H539E that had a substitution at the C-terminal ligand-binding domain [16], [45] (Fig. 1B) or in cells transfected with the empty vector (data not shown), demonstrating that the observed responses are from TLR3. The results from 293T cells are consistent with those from the BEAS-2B cells and support our observation that viral dsRNAs are poor agonists in comparison to poly(I:C).

To determine whether TLR3 was responsible for the increased IL6 levels, we treated cells with siRNAs specific to TLR3, RIG-I, or a nonspecific control for 48 h prior to the addition of BPEV dsRNA to the cells (Fig. 1C). RIG-I is a suitable control in this experiment since it also recognizes dsRNA [46]. Only cells treated with siRNAs to TLR3 had reduced IL6 levels in the presence of BPEV dsRNA (Fig. 1C). With poly(I:C)-induced cells, siRNAs to TLR3 also reduced IL6 production by 51±3% (n = 4) (data not shown; [18]). RT-PCR confirmed that the siRNAs did knock down TLR3 mRNA levels in both untreated and poly(I:C) treated cells (Fig. 1D). ODN2006, which we previously shown to inhibit TLR3 signaling [47] reduced the IL6 levels in the presence of poly(I:C) to less than 20% of the control reaction (data not shown) while EGCG, an inhibitor of RIG-I [48] had no effect on IL-6 production in the presence of poly(I:C) (data not shown). Thus, while the viral dsRNAs are poor TLR3 agonists, the low level of cytokine production by BEAS-2B cells was primarily due to the activation of TLR3. These results form the basis of the hypothesis to be tested in this work: the activation of TLR3 by viral dsRNA requires additional factors.

### Several viral dsRNA-binding proteins can enhance poly(I:C)-dependent signaling

Addition of the peptide LL37 to the cell culture media along with poly(I:C) or viral dsRNAs was found to enhance signaling by TLR3 [18]. LL37 binding to the dsRNA was correlated with enhancement of TLR3 signaling [18]. Since RNAs in cells exist in complex with proteins, we postulate that viral RNA-binding proteins could also enhance the TLR3 response to the viral dsRNAs. Several highly purified RNA-binding proteins were selected to test this idea. These include the recombinant Nsp15 protein from the SARS coronavirus that binds ssRNAs [49], two versions of the Hepatitis B virus capsid protein (H-cp149 and H-cp183) [38], the capsid protein from the Ross River virus (R-cp) [39], the capsid from the plant-infecting Brome mosaic virus (B-cp) [50] and polymerases from the 1b and 2a genotypes of the Hepatitis C virus (1bΔ21 and 2aΔ21) [27]. Except for the B-cp, which was extracted from cesium-chloride purified BMV virions, the other proteins were produced in *E. coli*.

**Figure 2:**
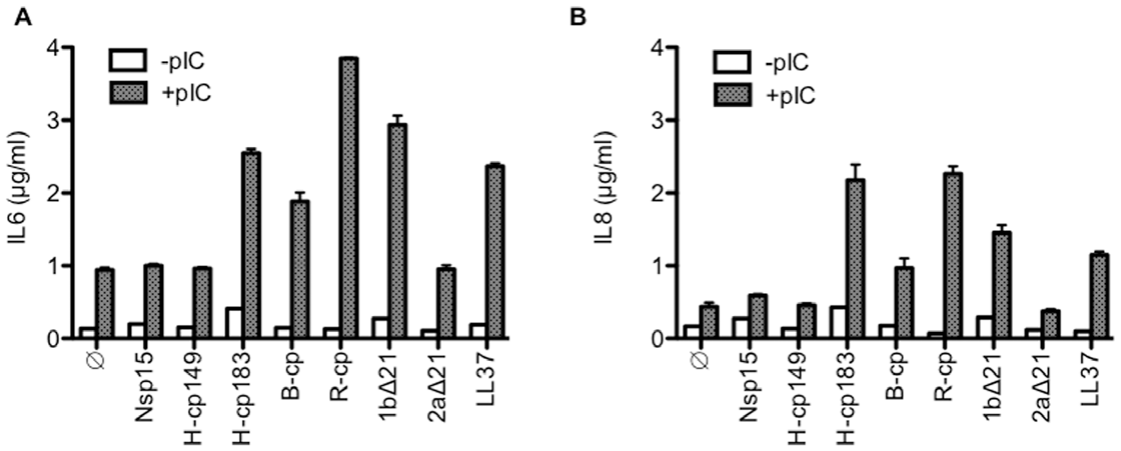
Several recombinant proteins can enhance cytokine production by poly(I:C). A) Quantification of IL6 production by BEAS-2B cells in response to the presence of various viral proteins with or without poly(I:C) at a final concentration of 0.13 µg/ml. Each protein was present at a final concentration of 0.15 µM. The antimicrobial peptide LL37 was added to 3 µM final concentration. IL6 was quantified in an aliquot of clarified cell medium and the total amount of IL6 was normalized to the total medium volume. The standard errors were from at least three replicates. B) A representative analysis of IL8 production by BEAS-2B cells in response to the exogenously added proteins. The conditions for the assay are identical to those in panel A.

While the cytokine induction by the various RNA-binding proteins in the absence of poly(I:C) varied depending on individual experiments, fold inductions of IL6 above basal across multiple experiments showed no statistically significance in paired T-tests (Fig. 2 and Table S1). These results, including those from B-cp that was produced in plants also suggest that common contaminants such as lipopolysaccharides which would induce TLR4 signaling, is not a significant factor in the assays. Incubation of poly(I:C) with the corresponding buffers in which the proteins were purified also did not result in a significant change of cytokine production (data not shown). The presence of poly(I:C) (0.13 µg/ml) alone induced IL6 and IL8 levels at least five-fold above background (Fig. 2). Several of the proteins added to a final concentration of 0.15 µM along with poly(I:C) resulted in additional increases in IL6 and IL8 levels of two- to five-fold (Fig. 2A and 2B; Table S1). The level of enhancement by the recombinant proteins were comparable to that observed with LL37 (3 µM).

The viral proteins that did not significantly increase cytokine production include the polymerase from HCV genotype 2a (2aΔ21), the truncated HBV CP (H-cp149), and the SARS-CoV Nsp15 (Fig. 2A and 2B). The Nsp15 used in this experiment is a catalytic-inactive version of an endoribonuclease that binds and preferentially cleaves ssRNAs [49]. Identical results were obtained with the WT Nsp15, hence the enzymatic activity of Nsp15 is not a factor in the response (data not shown). These results show that a subset of the RNA-binding proteins is more competent in enhancing TLR3 signaling. The activities of the HCV polymerases and the viral capsids will be analyzed in turn below.

### The HCV 1b polymerase can enhance dsRNA-induced signaling by TLR3

**Figure 3:**
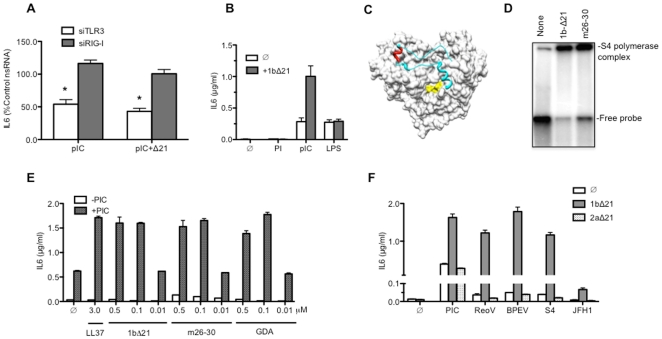
The 1b HCV polymerase can enhance IL6 production by BEAS-2B cells. A) Enhanced IL6 production in the presence of 1bΔ21 can be knocked down by SiRNA to TLR3. siRNAs specific to TLR3 (siTLR3), RIG-I (siRIG-I), or a nonspecific control (nsRNA) were transfected into at a concentration of 30 nM into cells 48 h prior to the addition of 1bΔ21 (0.15 µM) and poly(I:C) (0.13 µg/ml) to the culture medium. IL6 in the medium was collected 24 h later and quantified by ELISA. The data from 3 to 4 sets of samples are presented as a percentage of the IL6 in the sample treated with nsRNA (100%). B) IL6 production by BEAS-2B cells in response to stimulation by the single-stranded polyinosinic acid (0.13 µg/ml), poly(I:C) (0.13 µg/ml) or lipolysaccharide (LPS) (1 µg/ml), the agonist for TLR4 in the absence (Ø) or presence of 1bΔ21 (0.15 µM). C) A model of the 1b HCV polymerase that illustrates the location of the Δ1 loop (in light blue), the GDD active site (in yellow), and the short helix deleted in mutant m26–30 (in red). D) The 1bΔ21 polymerase and 1b.m26–30 can form a complex with the double-stranded S4 RNA. The S4 dsRNA was labeled with α-^32^P-CTP and used in an electrophoretic mobility shift assay. The gel image shown is from a non-denaturing stacked polyacrylamide gel of 5 and 20%. E) Effects of mutations in 1bΔ21 on IL6 production induced by poly(I:C). f denotes a reaction with no added proteins. Proteins 1bΔ21, 1b.m26–30, and the active site mutant GDA were added to the culture media (final concentrations 0.01, 0.1 or 0.5 µM) in the absence or presence of 0.13 µg/ml poly(I:C). LL37 was added to a final concentration of 3 µM. F) The 1b HCV polymerase (1bΔ21, 0.1 µM), but not the 2a polymerase (2aΔ21, 0.1 µM), allowed TLR3 to produce cytokines in response to viral dsRNAs. In addition to poly(I:C), the RNA ligands used were those extracted from Reovirus virions (ReoV), from the endornavirus BPEV, and the annealed transcripts of the sense and antisense strands made from the S4 cDNA of Reovirus (S4), and the transcript of the HCV 2a JFH-1.

We seek to determine whether the observed IL6 production in the presence of the 1b HCV polymerase was due to signaling by TLR3. SiRNAs to TLR3, to RIG-I, or to a nonspecific target were transfected into BEAS-2B cells prior to the addition of either poly(I:C) alone or poly(I:C) and 1bΔ21 (Fig. 3A). Only the cells transfected with the siRNAs to TLR3 had reduced IL6 production compared to cells transfected with nsRNA (Fig. 3A; p<0.05). We reproducibly observed using RT-PCR that TLR3 mRNA treated with the siRNAs were reduced to between 8 and 26% of the level in cells treated with the nsRNA control (Table S2). The presence of the polymerase did not change LPS-induced TLR4 response or change the response from cells treated with only polyinosinic acid, suggesting specific induction of TLR3 (Fig. 3B). Moreover, ODN2006, an inhibitor of TLR3 signaling [47] reduced IL6 levels to 40±7% of control while EGCG, an inhibitor of RIG-I [48] had no effect, further suggesting that the enhancement of dsRNA induced signaling is dependent on TLR3 (data not shown). The HCV polymerase also enhanced the response to poly(I:C) lengths that ranged from 45 to 125 bp (Fig. S2A). These results indicate that TLR3 is required for 1bΔ21 and poly(I:C) to enhance cytokine production.

1bΔ21 is less active for RNA synthesis *in vitro* than is the 2aΔ21 [27], [29], [30]. Therefore, it is unlikely that RNA synthesis by 1bΔ21 is responsible for the induction of TLR3 signaling. However, to establish this directly, we tested a catalytic mutant named GDA wherein the divalent metal-binding motif required for polymerization was mutated. We also tested three additional mutants that are defective for *de novo* initiation, including a five-residue deletion of the Δ1 loop name m26–30 [26]. The locations of the key mutations in the 1b polymerase structure are shown in Fig. 3C. Since m26–30 has a major change in conformational to 1bΔ21, we tested it for the ability to bind a dsRNA of ca. 900 bp derived from Reovirus genome and found that retained the ability to bind dsRNA (Fig. 3D). All of these mutant polymerases enhanced IL6 production over the level of poly(I:C) alone by at least two-fold (Fig. 3E and Fig. S2B).

Next, we examined whether 1bΔ21 could enhance IL6 production in concert with viral RNAs (Fig. 3F). In the absence of 1bΔ21, the single-stranded JFH-1 RNA and three different viral dsRNAs were poor inducers of IL6 production in BEAS2B cells when compared to poly(I:C). This result is consistent with our initial observation that TLR3 does not respond efficiently to viral RNAs (Fig. 1A & 3F, Fig. S1A). 1bΔ21 (0.1 µM) increased IL6 levels induced by the S4 dsRNA and the BPEV dsRNA by 4 to 6-fold (Fig. 3F). 1bΔ21 had only a modest increase when JFH-1 ssRNA was used as a ligand. In contrast, the activation of signaling was approximately 20-fold higher with dsRNA than with JFH-1 (Fig. 3F). Consistent with our previous observation, 2aΔ21 (0.1 µM) was unable to enhance IL6 production when added to cells along with either the ssRNA or dsRNA (Fig. 3F).

### The conformation of 2aΔ21 can affect dsRNA-dependent TLR3 signaling

**Figure 4:**
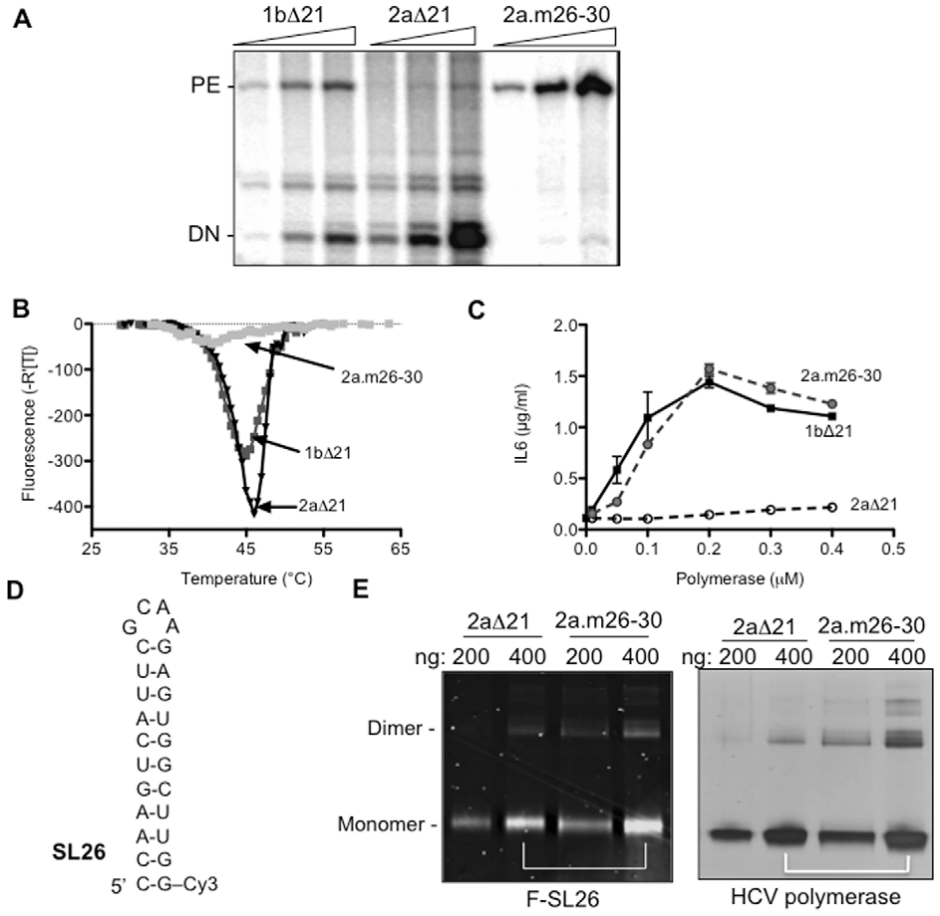
A mutant HCV 2a polymerase can enhance TLR3 signaling. A) *In vitro* RNA synthesis by the 1bΔ21, the 2aΔ21 and the 2a mutant 2a.m26–30. The final concentrations of proteins were at 40, 80, and 160 nM. The templates used, LE19P and PE46, can, respectively, produce de novo initiated products (DN) as well as those extended from a partial duplex RNA (labeled as “PE”) as described in Chinnaswamy et al. [27]. B) The denaturation profile of the proteins (5 µM) analyzed by differential scanning fluorimetry. The reactions were performed in a Stratagene Mx3005P real-time PCR machine in the presence of SYPRO orange, which fluoresces when complexed to denatured proteins. The ramp for temperature increase was at 0.5°C per min. C) The mutant HCV 2a polymerase, 2am26–30, can enhance poly(I:C) (0.13 µg/ml)-induced IL6 production by BEAS-2B cells. D) The sequence and likely structure of a fluorescently-labeled RNA, SL26, used to analyze polymerase-dsRNA interaction. E) Binding of SL26 by the three HCV polymerases. The proteins were mixed together with a 1∶1 molar ratio of SL26 then crosslinked with a Stratalinker prior to analysis by SDS-PAGE. The gel image on the left shows the fluorescently-labeled RNA shifted above the free SL26 at the bottom of the gel. The complexes that contain a monomer or a dimer of the HCV polymerase are labeled to the left of the gel image. The same gel was then stained with Coomassie blue to allow visualization of the proteins, the image of which is shown to the right.

2aΔ21 differs from 1bΔ21 in the relative amounts of product generated from *de novo* initiated RNA synthesis or extension from a primed template (Fig. 4A). 2aΔ21 produced high levels of *de novo* initiated RNA when compared to the primer extension products while 1bΔ21 produced similar amounts of the two products (Fig. 4A). In this assay, *de novo* initiated RNA synthesis was determined with template LE19P, whose 3′ terminal puromycin prevents primer extension, while primer extension used template PE46, which contains a partially double-stranded hairpin structure that can be extended by the polymerase. The products of the two modes of RNA synthesis demonstrate whether a single or a partially duplexed RNAs are bound in the template channel of the polymerase [24]–[26]. Furthermore, products from the two templates are associated with two distinct polymerase conformations: a structurally closed conformation can initiate better by a *de novo* mechanism or a more open conformation that favors primer extension [26]. Notably, the crystal structure of 2aΔ21 has a more closed conformation than that of 1bΔ21 due to additional interactions between the Δ1 loop and the thumb subdomain of the polymerase [29].

If the closed conformation of 2aΔ21 decreases its ability to bind dsRNA and to induce TLR3, a five-residue deletion at the tip of the Δ1 loop which results in a more open conformation should increase dsRNA binding and thus the RNA synthesis from a primed template. A protein with such a deletion named 2a.m26–30 was found to be debilitated in de novo initiated RNA synthesis while retaining the ability to extend from a primed template (Fig. 4A). 2a.m26–31 also showed a distinct thermal denaturation profile compared to that of 2aΔ21, when analyzed by differential scanning fluorimetry, consistent with the two proteins having different conformation(s) (Fig. 4B). Protein 2a.m26–30 added to BEAS-2B cells had no effect on IL6 production (Fig. 4C). When added along with poly(I:C) to BEAS-2B cells, 2a.m26–30 dramatically enhanced IL6 production to levels comparable to reactions containing 1bΔ21 (Fig. 4C, n = 4). These results clearly demonstrate that the conformations of the HCV polymerase that impact interaction with single or double-stranded RNAs affect the ability of the polymerase to enhance signaling by TLR3.

The deletion of the Δ1 loop in the 1b polymerase makes the template channel more accessible to dsRNAs [27]. To determine whether 2a.m26–30 was altered in RNA binding in comparison to 2aΔ21, we performed a UV-crosslinking assay with a fluorescently-labeled RNA SL26, which contains a GNRA tetraloop with an 11-bp stem (Fig. 4D). The 26-nt SL26 was used to minimize non-specific interaction by the polymerase so as to better analyze RNA binding potential of polymerase mutant. The RNA-protein complex was visualized with a phosphorimager and the position of the protein identified by staining with Coomassie blue (Fig. 4E). The purified HCV polymerase usually exists as a mixture of monomers and higher order oligomers [27]. Both the monomeric and dimeric forms of 2a.m26–30 bound SL26 better than those from 2aΔ21 (Fig. 4E). Taken together, these results show that the changes in 2a polymerase to increase its binding to a largely dsRNA is correlates with increased enhancement of TLR3 signaling.

### Cellular localization of 1bΔ21

**Figure 5:**
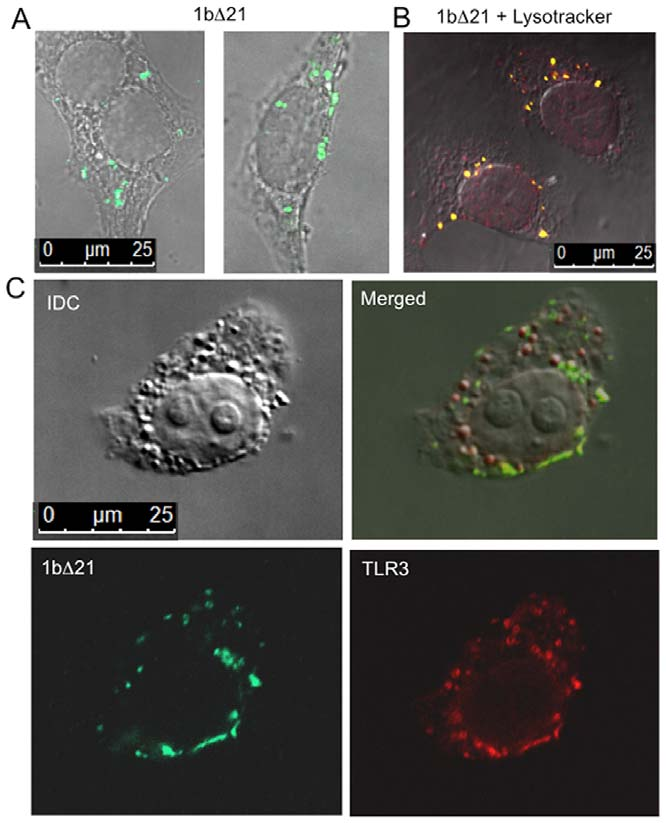
The HCV polymerase 1bΔ21 co-localizes with TLR3 in endosomes. A) Location of 1bΔ21 in BEAS-2B cells. 1bΔ21 (0.2 µM) was added to the medium of cells for 1 h and then detected by a mAb from Alexis Biochemicals. The locations of 1bΔ21 were then superimposed on a brightfield image of the same cells. B) NS5B is localized to acidic compartments in cells. BEAS-2B cells were stained with Lysotracker (Invitrogen, Inc). Lysotracker is colored red and sites of overlap between 1bΔ21 and lysotracker are in yellow. C) 1bΔ21 co-localizes with TLR3. A confocal section of a cell immunostained to localize both TLR3 and 1bΔ21, as well as the comparable brightfield are shown in the merged image. The yellow color identifies positions where the TLR3 and 1bΔ21 co-localized.

We sought to determine whether the recombinant HCV polymerase could enter cells and co-localize with TLR3 in endosomes. 1bΔ21 added to the medium of BEAS-2B cells in the absence of poly(I:C) was localized to punctate spots within 30 min. of addition (Fig. 5A). Some of these spots co-localized with Lysotracker that stains acidic compartments in the cell where TLR3 is located (Fig. 5B; [15]). The observation that not all of the dsRNA co-localized with TLR3 is consistent with previous reports that dsRNA could traffic into cells independent of TLR3 [47], [51].

To determine whether 1bΔ21 co-localized with TLR3, cells exposed to 1bΔ21 for 6 h were stained with the antibodies to TLR3 and the HCV polymerase. Signals to both proteins overlapped significantly, although not at every location where TLR3 was detected (Fig. 5C). The co-localization of the HCV polymerase to endosomes containing TLR3 may factor into the mechanism to enhance TLR3 signaling.

The localization of 1bΔ21 in BEAS-2B cells suggests that it is taken up by endocytosis. Arginine-rich cell penetrating peptides and Vaccinia virus had been reported to enter cells by several pathways, including macropinocytosis [52], [53], [54]. To examine whether macropinocytosis is likely involved in the uptake of 1bΔ21, we tested the inhibitor 5(N-ethyl-N-isopropyl)-amiloride (EIPA) on TLR3 signaling in the presence of poly(I:C) and either LL37 or recombinant 1bΔ21 (Fig. S3). EIPA dose-dependently inhibited TLR3 signaling by either poly(I:C) alone, or poly(I:C) added to the cell culture medium along with LL37 or 1bΔ21. These results are consistent with macropinocytosis playing a role in the uptake of either the dsRNA and/or the recombinant protein into endosomes.

### Capsid proteins and TLR3 signaling

**Figure 6:**
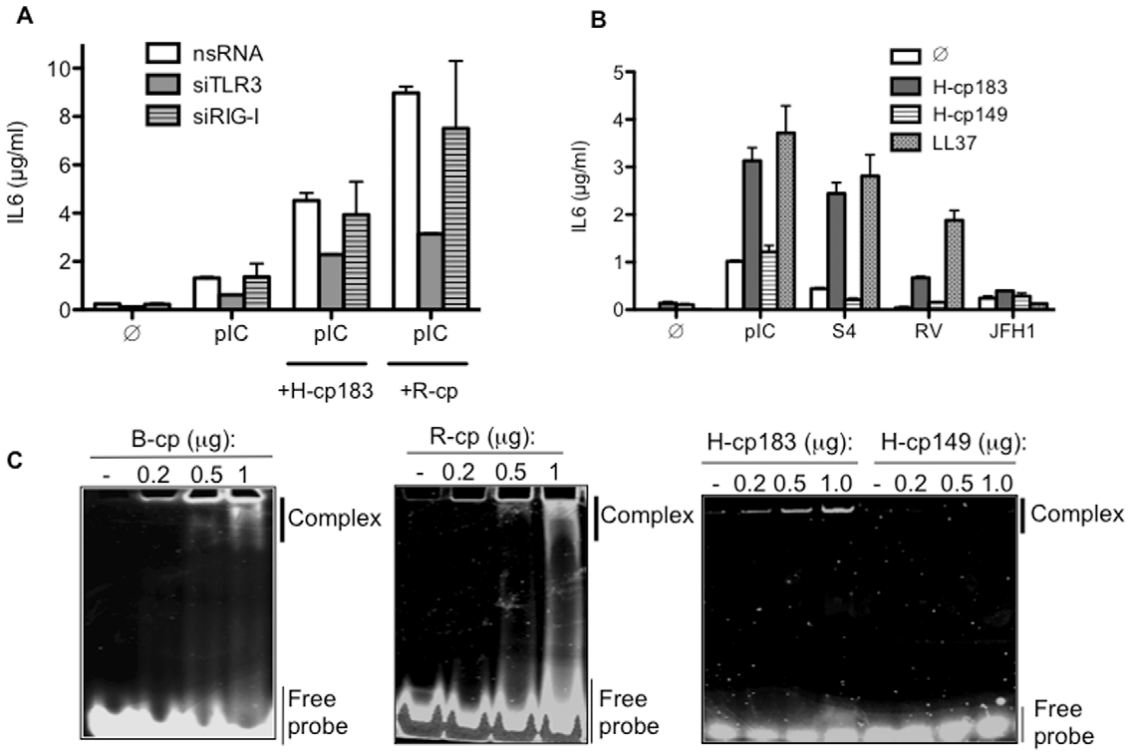
Effects of three viral capsids on IL6 production by BEAS-2B cells. A) Enhancement of IL6 production by the viral capsids requires TLR3. SiRNAs to TLR3, siRNA to RIG-I, or a nonspecific target were transfected at 30 nM into the cells 48 h prior to the addition of the viral capsid proteins (50 nM) either alone or in combination with poly(I:C) (0.13 µg/ml). B) Effects of H-cp183 or H-cp149 (50 nM) and viral dsRNAs on the production of IL6. The reactions with LL37 serve as a positive control. DsRNA S4 is a mimic of the shortest of the Reovirus dsRNAs (ca. 1100 bp) made by annealing *in vitro* transcripts of the plus- and minus-sense RNAs. RV denotes the total virion RNA purified from reovirus particles. JFH-1 is the *in vitro* transcript of the genotype 2a HCV. C) Effects of increasing B-cp, R-cp, H-cp183, and H-cp149 on the ability to bind and crosslink to SL26. The crosslinking reactions were electrophoresed on SDS-PAGE and imaged on a Phosphorimager.

To extend the analysis of viral protein-RNA interaction further, we characterized the effects of three capsid proteins (CPs) that enhanced the dsRNA-specific innate immune signaling (Fig. 2). Knockdowns of TLR3, RIG-I, or a nonspecific target prior to stimulation with poly(I:C) and the CPs (50 nM) showed that TLR3 was the receptor primarily responsible for IL6 production for H-cp184 and R-cp (Fig. 6A). SiRNAs to TLR3 also decreased B-cp induced IL6 production (data not shown). In addition, all three WT viral CPs increased IL6 production in a concentration-dependent manner (Fig. S4). H-cp149 that lacks the C-terminal sequence of H-cp183 (Fig. S4A) was unable to modulate IL6 production (Fig. S4B).

We examined whether the CP can enhance signaling by viral ssRNA or dsRNA. IL6 level were enhanced when the Reovirus genomic RNAs and the S4 dsRNA (all at 0.15 µg/ml) were added to the medium in the presence of H-cp183. The single-stranded JFH-1 RNA was not significantly enhanced by H-cp183. Furthermore, H-cp149 did not result in statistically significant enhancement of IL6 in the presence of viral dsRNAs or the JFH1 ssRNA (Fig. 6B, Fig. S4B). The amount of enhancement with the 150 nM of H-cp183 was comparable to that of 3 µM LL37. The three viral CPs thus can enhance TLR3 signaling in response to both poly(I:C) as well as viral dsRNAs.

### RNA-binding is required to enhance dsRNA-dependent signaling by TLR3

The differential effects of H-cp183 and H-cp149 should reveal a requirement important for the enhancement of dsRNA-dependent signaling by TLR3. The HBV CP contains sixteen arginines in its C-terminal thirty-four residues (Fig. S4A). H-cp149 is competent for the formation of virus-like particles, but is defective for binding to RNA [38]. It is therefore likely that the altered interaction with RNA is responsible for the inability of H-cp149 to enhance TLR3 signaling when compared to the other capsid proteins. Again, to reduce the effects of nonspecific RNA binding inherent to many RNA-binding proteins, SL26 containing an 11-bp dsRNA was used to examine whether H-cp183 and H-cp149 are distinguishable in their ability to bind RNA. SL26 bound H-cp183 as well as B-cp, and the R-cp (indicated by shifts to higher molecular mass smears). However, no shift occurred with H-cp149 (Fig. 6C). These results provide another set of example wherein a viral protein's ability to bind dsRNA is linked to the enhancement of TLR3-dependent signaling.

### The RRV CP can enter cells and co-localize with TLR3 in endosomes

**Figure 7:**
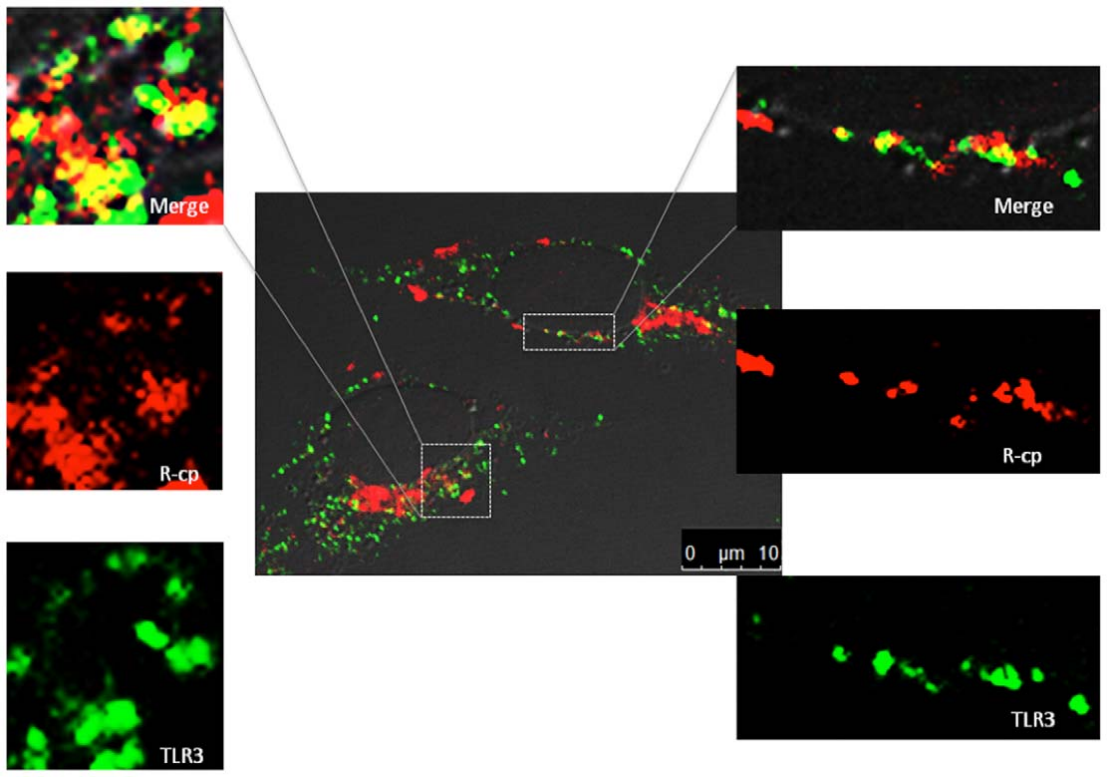
The RRV CP can enter cells and co-localize with TLR3. A) A merged brightfield image of BEAS-2B cells and the locations of the R-cp. The R-cp was added to the cells for 2 h prior to processing the cells for immunolocalization. R-cp antibody was custom made by Cocalico Biologicals (Reamstown, PA). The boxed area in the upper right corner is increased in size in the lower figure to allow better visualization of the punctate spots of R-cp within the cell. Co-localization of the R-cp with TLR3 is shown in the magnified images. TLR3 is imaged in blue and R-cp in red and locations of co-localization in yellow.

The HCV polymerase 1bΔ21 co-localized with TLR3 in endosomes (Fig. 5). The BMV CP has also been shown to enter barley cells through macropinocytosis and localize to punctate spots within the cytoplasm that are suggestive of endosomes [53]. The HBV capsid can also enter cells, although permeabilization of the cells with digitonin was required [55]. We seek to determine whether the capsid proteins can localize to endosomes that contain TLR3, using the R-cp as an example. 2 h after the addition of R-cp to the media of BEAS-2B cells, R-cp was found close to the plasma membrane, with a small proportion of the signal localizing in endosomes (Fig. S5). At 6 h after R-cp addition, the majority of the signal was within endosomes (Fig. 7). Immunostaining to detect TLR3 revealed an extensive co-localization of R-cp (Fig. 7).

### RNA-binding proteins can enhance viral dsRNA-dependent signaling in HEK 293T cells

**Figure 8:**
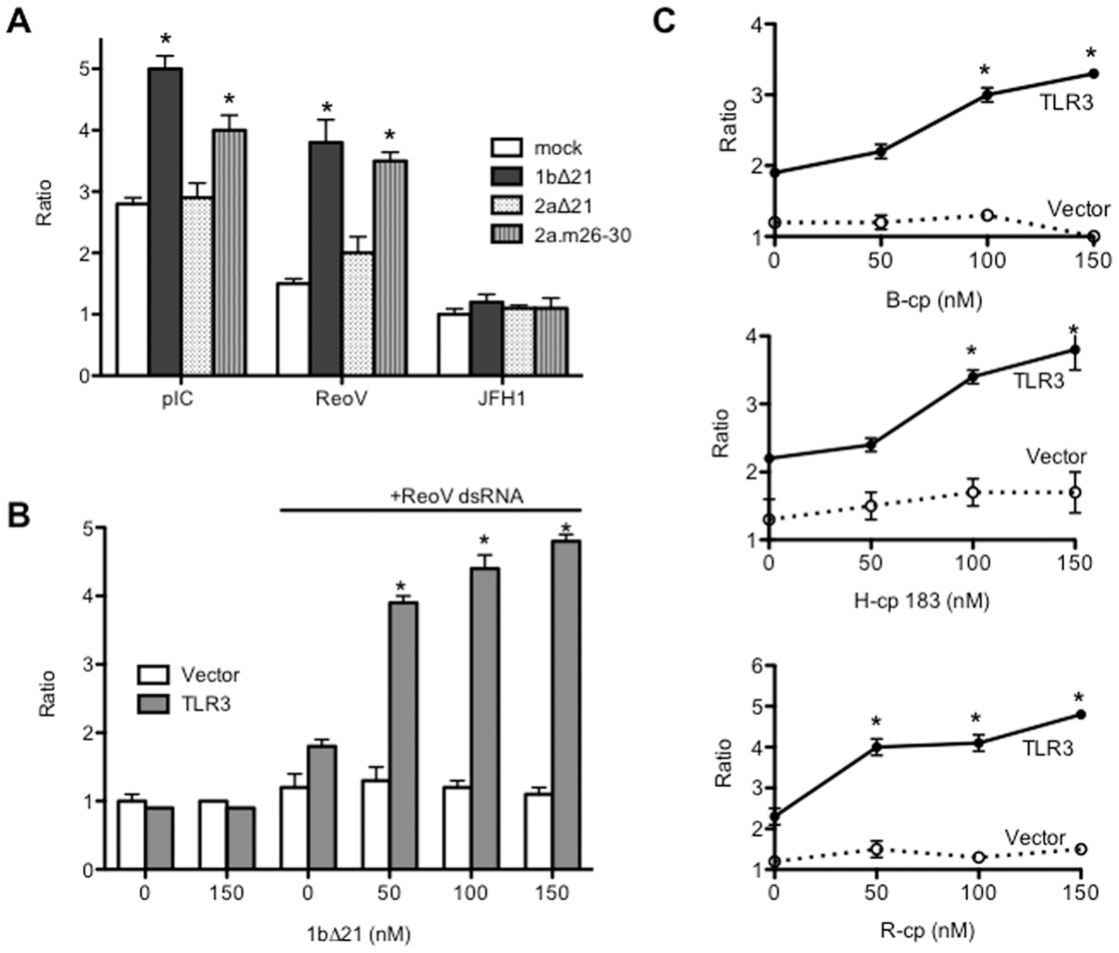
Viral proteins can enhance TLR3-dependent reporter expression in HEK 293T cells. A) The effects of HCV polymerases on ISRE-luciferase expression in 293T cells transiently-transfected with TLR3. All recombinant proteins used were added to cell culture media to a final concentration of 100 nM. The ratio of the firefly luciferase levels to the *Renilla* luciferase is shown on the Y-axis. B) The recombinant 1bΔ21 protein (0 to 150 nM) concentration-dependently increased the ratio of the firefly to *Renilla* luciferases induced by RV dsRNA (1 µg/ml). C) Purified CPs can enhance TLR3-dependent signaling from RV dsRNA in a concentration-dependent manner. The ratios are from ISRE-dependent firefly luciferase levels over the *Renilla* luciferase. The concentrations of the CP used in the assay are shown on the horizontal axes. The mean values of at least three independently assayed samples are shown above the bars.

The effects of the HCV polymerases and the viral capsid proteins have thus far been examined in BEAS-2B cells. We seek to determine whether they could enhance signaling in HEK 293T cells transiently transfected to express TLR3. TLR3-specific activation of luciferase production from an ISRE promoter element was used to assess signal transduction. Reporter production in the presence of poly(I:C), and Reovirus genomic dsRNA were all reproducibly enhanced in the presence of 1bΔ21 or 2a.m26–30, but not with 2aΔ21 (Fig. 8A). As with the BEAS-2B cells, JFH1 RNA was unable to induce TLR3 in HEK 293T cells. The activation of reporter production was dependent on the concentration of 1bΔ21 added to the 293T cell culture media (Fig. 8B). The effects of the viral CPs on TLR3 signaling in HEK cells were also examined using the Reovirus dsRNA as the TLR3 ligand. B-cp, H-cp183, and the R-cp all increased reporter levels in a concentration-dependent manner when compared to cells transfected with only the empty plasmid vector (Fig. 8C). This result shows that all five recombinant proteins that enhanced TLR3 signaling with viral dsRNAs in BEAS-2B cells enhanced TLR3-dependent signaling in HEK 293T cells.

## Discussion

While TLR3 binds to dsRNA to activate innate immune responses, viral dsRNAs tend to be poor TLR3 agonists, especially when compared to the synthetic dsRNA analog, poly(I:C). In a companion manuscript [18], we demonstrated that LL37 is a potent enhancer of dsRNA-dependent TLR3 signaling. Herein, we demonstrate that a subset of viral proteins can significantly enhance both poly(I:C) and viral dsRNA-induced TLR3 signaling. These viral proteins include polymerases from two genotypes of hepatitis C virus and three viral capsid proteins. Requirements for the polymerases and capsid proteins (1bΔ21, 2a.m26–30, B-cp, R-cp, and H-cp183) to activate TLR3 signaling were examined and a common property is the ability to bind dsRNAs. Two proteins (2aΔ21 and H-cp149) that bind dsRNA less well are also less effective in enhancing TLR3 signaling. These results suggest that features that affect dsRNA binding modulate the ability of proteins to activate TLR3 signaling. A second requirement elucidated with the 1b HCV polymerase and the RRV CP is the ability of the proteins to enter cells and co-localize with TLR3 in endosomes. The focus of the discussion below will be on the requirements for activating TLR3 signaling, with a final section on the conformation of the HCV polymerase.

### Agonists for TLR3

The chemical features in dsRNA that are recognized by TLR3 are poorly understood. The difference between what makes poly(I:C) a good TLR3 agonist in the absence of RNA-binding proteins and what makes the viral dsRNAs inferior ones will require in-depth analysis. We believe that this work identifies conditions that will facilitate these efforts. Based on our current results, Reovirus S4 dsRNA made by *in vitro* transcription is as potent a TLR3 agonist as the genomic dsRNA extracted from Reovirus virions, suggesting that no obvious posttranscriptional modifications are required to activate TLR3 signaling. Another feature of poly(I:C) that may obviate the need for viral proteins or LL37 may be that the inosine-cytosine base pair has only two H-bonds. We observed that poly(A:U) is also an excellent agonist for TLR3 in the absence of LL37 and viral protein (Fig. S1A). However, weaker base pairing alone is unlikely to provide the full explanation for poly(I:C) being such a potent agonist; poly(G:U), which also has two H-bonds per base pair, is a poor agonist for TLR3 signaling in the absence of viral dsRNA proteins (Fig. S1A).

We can offer the following observations on proteins and peptides that can enhance TLR3 signaling. The proteins can be produced in *E. coli* or extracted from virions (as in the case of BMV), suggesting that post-translational modifications are not required for the enhancement of the TLR3 response. Similarly, peptides such as LL37 that are potent enhancers of TLR3 signaling can be synthesized chemically without specific modifications [18]. In addition, both enzymes and structural proteins can be potent enhancers of TLR3 signaling as long as they interact with either dsRNA or partially duplexed RNAs. The SARS-CoV Nsp15 preferentially binds ssRNA and not dsRNA [49], and could not efficiently enhance TLR3 signaling. A deletion that increased interaction between the 2a HCV polymerase and a partially duplexed RNA template made the polymerase a better enhancer for TLR3 signaling (Fig. 4C). The second feature of the TLR3 enhancers is that they have the ability to form oligomers. This is intuitive with the viral capsid proteins, but viral RdRps have surfaces that mediate oligomerization that may facilitate RNA binding [56], [57], [58]. Indeed, oligomerization of the HCV polymerase can affect the mode of RNA synthesis [27]. LL37 also exists in higher order complexes in the absence of RNA and is required at micromolar concentrations in contrast to the viral proteins that are active in our assays at nanomolar concentrations. This is likely due to multiple LL37 molecules being needed to bind and/or coat the dsRNAs and mediate TLR3 activation [18].

The fact that BMV and RRV encapsidate ssRNAs may initially seem to incompatible with their ability to enhance signaling by dsRNAs. However, the encapsidated viral RNAs are highly compacted and contain a high percentage of dsRNAs with non-Watson-Crick base pairing, close to 70% [59], [60]. This likely explains how the CP can bind the dsRNA needed to activate TLR3. The CP's of BMV and RRV are capable of binding to the double-stranded S4 RNA in an electrophoretic mobility shift assay (Fig. S4C). In the context of a viral infection, however, we note it is likely that fully assembled virions may not result in activation of TLR3 signaling if the dsRNAs are not exposed. We have added highly purified BMV virions and Reovirus virions to BEAS-2B cells and 293T cells expressing TLR3 and have not observed induction of cytokines or TLR3-specific reporters (Kao and Ranjith-Kumar unpublished results).

We also demonstrated that two dsRNA-binding proteins that can enhance TLR3 signaling can colocalize with TLR3 in endosomes. Whether the dsRNA-binding proteins facilitate binding of the dsRNA by the TLR3 or the uptake of dsRNA to endosomes remain to be determined. However, it is known that TLR3 binds to linear dsRNA [61]. TLR3, unlike the RIG-I-Iike receptors [62], lacks helicase activity to unravel the tertiary interactions that could stabilize the dsRNA. The dsRNA-binding proteins could disrupt the tertiary structure in the dsRNAs to facilitate recognition of the dsRNA by TLR3. Electron microscopy revealed that LL37 decreases the particle size of the primarily globular poly(I:C) and results in more filamentous structures [18]. With the second scenario, the dsRNA-binding proteins could facilitate entry of the viral dsRNAs into endosomes, perhaps by increasing the interaction with trafficking chaperones, such as scavenger receptors [51], but these RNA-binding proteins likely need to complex and bring dsRNA to endosome in order for TLR3 activation to occur. The net effect is an increase in the concentration of ligand available to TLR3. Further studies will be required to unravel the complex requirements whereby the dsRNA-binding protein activate TLR3 signaling.

Our demonstration that LL37 and viral dsRNA-binding proteins can enhance TLR3 signaling raises the possibility that inappropriately expressed cellular dsRNA-binding proteins could activate TLR3 signaling from cellular RNAs. Autoimmunity has been linked to antibodies that recognize RNP complexes [63], [64], and TLR signaling can affect the production of autoantibodies associated with lupus erythematosis [65].

### The HCV polymerases

The results in this work are also informative for the function of the HCV polymerase. We note that the enhancement of TLR3 signaling by HCV polymerase is independent of RNA synthesis since the active site mutant is capable of enhancing TLR3 signaling (Fig. 3D). All polymerases undergo structural transitions between open and closed conformations in response to the stages of nucleic acid synthesis. Indeed, the monomer of the HCV polymerase contains a template channel that can sterically accommodate ssRNA, but not dsRNA [26], [66]. During the formation of a nascent RNA from the template, however, the polymerase needs to release the interaction between the Δ1 loop and the thumb subdomain in order to accommodate the elongating nascent RNA-template RNA duplex. The 1bΔ21 can both extend from a primed template as well as initiate from the 3′ terminus of a single-stranded RNA. We believe that this is a manifestation of the 1b polymerase existing in an equilibrium between the open and closed forms *in vitro*, likely accounting for better dsRNA-binding by the 1b polymerase and its enhanced ability for dsRNA-induced signaling by TLR3. In contrast, the 2a polymerase exists predominantly in a closed conformation and thus is less able to bind to dsRNA and enhance signaling by TLR3 [29]. Increasingly, the open and closed conformations of the HCV polymerases are being linked to polymerase function. For the 1b HCV polymerase, the open conformation also exposes a binding pocket for the cell cycle regulator retinoblastoma and mediates the ubiquitinylation and degradation of retinoblastoma to increase cell cycle progression during HCV infection [67], [68].

During viral infection, viral polymerases and RNAs are expressed inside the cell and depending on the cellular locations of the polymerase protein and viral or cellular dsRNAs they recognize, could activate cytoplasmic innate immunity receptors such as RIG-I or endosomal TLRs. Previous study by Naka et al [69] found that immortalized liver cells stably transfected with HCV NS5B and HEK 293 cells stably transfected with both NS5B and TLR3 genes show increased activation of TLR3-depedent IFNβ signaling, although the activation in HEK 293 cells was only 2-fold. In a recent study from our laboratory, transient overexpression of NS5B and RIG-I resulted in 8 to 14-fold induction of IFNβ promoter-driven luciferase activity in Huh7 and in HEK 293T cells while overexpression of NS5B and TLR3 had no effect, suggesting that expression of NS5B in these two cell lines predominantly activates cytoplasmic innate immunity receptors such as RIG-I and MDA5 [70]. In both these studies, the NS5B polymerase was expressed from transfected plasmids and catalytic activity of the polymerase is required. In the present study, exogenously added HCV polymerase protein activates TLR3 without requiring RNA synthesis since polymerase mutated at the catalytic site still activates TLR3. In addition three viral capsid proteins as well as LL37 that bind RNA but do not have catalytic activity also activate TLR3 signaling. Addition of both dsRNA and the RNA-binding protein are required for this activation. RNA-binding protein alone has no effect since RNAs synthesized by the HCV polymerase in cells may not enter efficiently into acidic endosomes, the site of TLR3 signaling [15]. Thus the effect and mechanism of TLR3 activation by RNA-binding protein are likely different from that observed by Naka et al. [69] or Ranjith-Kumar et al. [70].

During viral infection and subsequent cell lysis, viral proteins and RNAs may be released to the cell media and these viral proteins and RNAs may be taken up by cells to activate innate immunity. Exogenously added HCV core and NS3 proteins enters cells to activate TLR2 signaling in the absence of any added ligand, although the mechanism(s) of activation is not known [71]. In the present study, addition of NS5B protein to BEAS2B or HEK293T cells has minimal effect on its own but significantly activates TLR3 signaling in combination with dsRNA ligands, including viral dsRNAs (Table S1). Indeed, NS5B and the RRV capsid protein have the ability to enters BEAS-2B cells within 30 min and colocalize with endosomal TLR3 by 6 h. The cellular entry of released viral proteins and RNAs during viral infection may contribute to the cellular innate immune responses and could provide a point for therapeutic intervention.

## Materials and Methods

### Reagents

Recombinant genotype 1b (Con1 strain) and 2a (JFH-1) HCV polymerases lacking the C-terminal transmembrane helix were purified from *E. coli* as described by Chinnaswamy et al. [28]. The preparations were devoid of detectable contaminating RNA, DNA, or LPS. The HBV and RRV capsid proteins were expressed in *E. coli* and purified according to Porterfield et al. [38] and Mukhopadhyay et al., [39]. Poly(I:C) was purchased from Amersham Biosciences (Piscataway, NJ) and reconstituted in phosphate-buffered saline. RNA from purified Reovirus virions was extracted with Trizol, followed by ethanol precipitation and a wash with 70% ethanol. The purified RNA was stored in RNase-free water. S4 dsRNA from Reovirus was synthesized by in vitro transcription from cDNA (kind gift of P. Danthi, Indiana University, Indiana) that has been engineered to have T7 promoters at termini of the two complementary strands. The annealed RNAs were then analyzed for quantity and concentration before use. BPEV dsRNA was purified according to the protocol of Valverde and Gutierrez [43] and stored in RNase-free water. BEAS2B and HEK293T cells were from ATCC and cultured in BEGM media with supplements (Lonza, Basel, Switzerland).

### Quantification of IL6 level in culture supernatant

Culture media from BEAS-2B cells were collected 24 h after treatments, centrifuged at 2000 g for 2 min, and the supernatants were assayed for IL6 secretion using a human IL6 ELISA kit along with quantification controls (BD systems) as per the manufacturer's protocol.

### TLR3 luciferase reporter assay

HEK293T cells were plated in CoStar white 96-well plates in DMEM amended with 10% FBS at 4.4×10^4^ cells per well and transfected at ∼85 to 90% confluency with a mixture of the Lipofectamine 2000 and plasmids pISRE-Luc, pUNO-huTLR3 and phRL-TK as described in Sun et al. [44]. The transfected cells were induced 18 to 22 h later by the addition of poly(I:C) or viral dsRNAs to the culture medium to a final concentration of 1 µg/mL in the presence or absence of 100 nM of RNA binding proteins. The cells were harvested for analysis of luciferase 14 to 18 h after poly(I:C) addition.

### RdRp assays

RdRp assays were performed as 20 µL reactions containing 20 mM sodium glutamate (pH 8.2), 12.5 mM DTT, 4 mM MgCl_2_, 1 mM MnCl_2_, 0.5% Triton X-100, 0.2 mM GTP, 0.1 mM ATP and UTP, 250 nM [α-^32^P] CTP (MP Biomedicals), 2 pmol PE46 and 1 pmol LE19p as templates [26]. The reaction mixture was incubated at 30°C for 1 h and terminated by phenol-chloroform extraction, followed by precipitation of the RNA in the presence of two volumes of ethanol, 5 µg glycogen and 0.3 M NaOAc (pH 5.2). RNA products were separated by 20%-7.5 M urea polyacrylamide gels. The signals were detected and quantified by using a PhosphorImager.

### UV cross-linking assay and gel shift assay

Each reaction contained 400 ng of purified protein mixed with 1 pmol Cy5-labeled SL26 or S4 dsRNA radiolabled by a polynucleotide kinase reaction in a buffer containing 50 mM Tris-HCl (pH 7.5), 4 mM MgCl_2_, 50 mM NaCl, and 1 mM dithiothreitol. The reaction was irradiated with UV at 1200 mJ for 3 min. and then subjected to SDS-PAGE. The signals were detected and quantified with a phosphorimager. The gel was then stained with Coomassie blue to visualize the locations of proteins.

### Differential scanning fluorimetry

DSF was performed in a Stratagene MX3005P Real-time PCR machine according to the protocol of Niesen et al. [72]. Each sample (5 μM final concentration) was prepared in a total volume of 50 μL containing SYPRO orange (Molecular Probes) in 100 mM Tris, pH 7.0, 50 mM KCl, and 5 mM MgCl_2_). The ramp condition was from 25 to 75°C, at increments of 0.5°C/min and the change in fluorescence intensity used to determine the *T*_m_ values with the use of KaleidaGraph Software (Synergy, Reading, PA).

### siRNA knockdown experiments

BEAS-2B cells were seeded at 1.0×10^5^ cells/well in BEGM amended with supplements (Lonza, Switzerland) in a 48-well tissue culture plate or 1.0×10^4^ cells/well in a 96-well plate. 6 h later, the cells were transfected with a pool of three siRNAs specific to TLR3, nonspecific control siRNA (Santa Cruz Biotechnology Inc.), or siRNA to RIG-I (Qiagen, Valencia CA). 30 nM SiRNAs were transfected using Lipofectamine RNAiMax (Invitrogen, Carlsbad CA) according to the manufacturer's protocol. The cells were incubated for 48 h prior to treatment with ligands and proteins. Culture media was collected 24 h later and assayed for IL6 production using ELISA. RT-PCR analysis of the TLR3 mRNA to confirm the effects of the siRNA knockdown was performed. After treatments total RNA samples pooled from either six wells of a 96-well plate or three wells of a 48-well plate were isolated from BEAS2B cells using RNeasy kit (Qiagen, Valencia, CA). 0.5 μg of total RNA was then reverse transcribed to cDNA with MMLV reverse transcriptase (Ambion, Austin, TX) using random decamers (New England Biolabs, Ipswich, MA). cDNA generated from 16 ng of total RNA was amplified using SYBR Green Supermix (Bio-Rad, Hercules, CA) with an initial 3 min denaturing temperature of 95°C, followed by a total of 40 cycles of 30 s of denaturation at 95°C and 30 s of annealing at 55°C and elongation at 72°C. TLR3-specific primers were previously used by Homma et al. [73]: (forward: 5′-GATCTGTCTCATAATGGCTTG-3′; reverse: 5′-GACAGATTCCGAATGCTTGTG-3′; and GAPDH primers (5′-GAGTCAACGGATTTGGTCGT-3′; reverse: 5′-TGGGATTTCCATTGATGACA-3′) were used. For each sample, TLR3 Ct values were subtracted from corresponding GAPDH Ct values. TLR3 mRNA levels for each treatment were calculated as %TLR3 mRNA for samples treated with control nonspecific siRNA.

### Fluorescence microscopy

BEAS-2B cells were seeded on coverslips in 6 well plates. Highly purified recombinant NS5B (0.2 μM) that was free of pyrogen was added to the cells at the same time as the dsRNAs and incubated for 30 min at 37°C. After washing with PBS, cells were fixed with 4% paraformaldehyde at room temperature for 30 min. After two additional washes with PBS, they were permeabilized with 0.5% Triton X-100 at room temperature for 10 min. The cells were then washed twice more with PBS amended with 0.02% Tween-20 (TBS-T) and incubated with blocking buffer (1% BSA in PBS) for 1 h at room temperature. After washing three times with PBS-T, slides were incubated with anti-NS5b antibody (mAb; 5B-3B1 from Alexis Biochemicals) and goat anti-TLR3 (R&D systems, Minneapolis, MN) overnight at 4°C. Slides were then probed with a Texas Red-labeled bovine anti-goat mAb (Santa Cruz biotechnology) for 1 h at RT, washed, then incubated with a goat anti-mouse secondary antibody conjugated with Alexa fluor 488 (Santa Cruz Biotechnology) for 1 h at room temperature. Coverslips were washed with PBS-T three times, air dried for 1 h and mounted in Vectashield mounting medium with DAPI (Vector Laboratories). The images were obtained on a Leica TCS SP5 scanning confocal microscope with an HCX PL APO Lambda Blue 63 X 1.4 oil objective lens (Leica Microsystems). Excitation was at 20% of the maximum laser power. Images were captured with a scanning speed of 200 Hz and image resolution of 512×512 pixels and then analyzed using Leica Application Suite 2.02.

Ross River virus capsid protein (0.2 μM) was added the culture media of BEAS-2B cells seeded on coverslips for up to 6 hours at 37°C. The cells were washed and fixed as described above. The cells were incubated with anti R-cp rabbit polyclonal antibody (custom prepared by Cocalico Biologicals, Reamstown, PA) and anti-TLR3 (mAb, a kind gift of L. San Mateo of Centocor Inc) diluted in PBS+0.1% BSA+0.01% TX100 overnight at 4°C. Slides were then probed with a goat anti-mouse secondary antibody conjugated with Alexa fluor 488 (Santa Cruz Biotechnology) for TLR3 and bovine anti-rabbit Texas Red (Santa Cruz Biotechnology) for R-cp for 1.5 h at room temperature. The slides were imaged and the results analyzed as described above.

## Supporting Information

Figure S1 **Effects of various RNAs on IL6 production.** A) Results from BEAS-2B cells. The endornaviral dsRNAs labeled A–C were extracted from several rice isolates collected in Lousiana and have distinct migrations in agarose gels. However, the genomes have not been determined. pIC, pAU, and pGU are three homopolymeric RNAs. The single-stranded viral RNAs were all extracted from purified virions. All RNAs used were at a final concentration of 0.5 µg/ml. B) A time course of IL6 production by BEAS-2B cells in response to poly(I:C) and S4 dsRNA. The addition of LL37 to 3 µM final concentration resulted in TLR3 enhancing signaling in response to the S4 dsRNA or poly(I:C). C) Results from HEK 293T cells expressing recombinant TLR3. All RNAs were used at 1 µg/ml. The ratios denote the ratio of the ISRE-driven firefly luciferase to the *Renilla* luciferase produces in the same cells.
(TIF)

Figure S2 **Additional characterization of the 1b HCV polymerase that could affect IL6 production in BEAS-2B cells.** A) Effects of poly(I:C) of different lengths on IL-6 production. The poly(I:C) fragments were separated on a denaturing gel and eluted from the gel fragments. The eluted fragments were annealed and their lengths determined by comparison to an DNA ladder. B) Effects of several recombinant HCV polymerase proteins on TLR3 induction of IL6. The final concentrations of the proteins added are shown on the horizontal axis. The amount of poly(I:C) in each reaction was at 0.13 µg/ml. C) A demonstration that the mutant HCV polymerases retain the ability to bind the SL26 RNA.
(TIF)

Figure S3 **Macropinocytosis may be involved in TLR3 signaling.** A) Effects of 5-(N-ethyl-N-isopropyl)-amiloride (aka EIPA) on TLR3 signaling. Where present, LL37 was at the final concentrations of 3 µM, 1bΔ21 or 2aΔ21 were at 0.5 µM, and poly(I:C) was at 0.13 µg/ml. B) Concentration-dependent inhibition of signaling by TLR3 in the presence of poly(I:C) and the cofactors.
(TIF)

Figure S4 **Additional information on viral capsids and their effect on TLR3 signaling.** A) The sequence of the hepatitis B virus capsid protein. The residues present in H-cp183 but absent in H-cp149 are underlined and the arginine residues are in blue. B) Concentration-dependent effects of the viral capsid proteins on TLR3 signaling. C) A demonstration that the capsids from BMV and RRV that have ssRNA genomes can bind the S4 dsRNA. The gel is from a nondenaturing stacked polyacrylamide gel of 5 and 20%. The S4 dsRNA was radiolabeled during in vitro transcription of the plus- and minus-strands and then annealed at a 1∶1 ratio.
(TIF)

Figure S5 **Localization of the R-cp in BEAS-2B cells 2 h after addition into the cell culture medium.** R-cp is stained red. The boxed area in the upper right is enlarged to show that small punctate spots of R-cp can be found in the cell's cytoplasm.
(TIF)

Table S1 **Effects of RNA-binding proteins on IL6 levels in BEAS-2B cells.**
(TIF)

Table S2 **Effects of siRNAs to TLR3 on TLR3 mRNA levels in BEAS-2B cells, as determined by RT-PCR.**
(TIF)
