# Supplementary figures and images for: Viral Double-Strand RNA-Binding Proteins Can Enhance Innate Immune Signaling by Toll-Like Receptor 3
Source: PLoS One. 2011 Oct 10;6(10):e25837. doi: 10.1371/journal.pone.0025837 (PMC3189932; doi:10.1371/journal.pone.0025837)

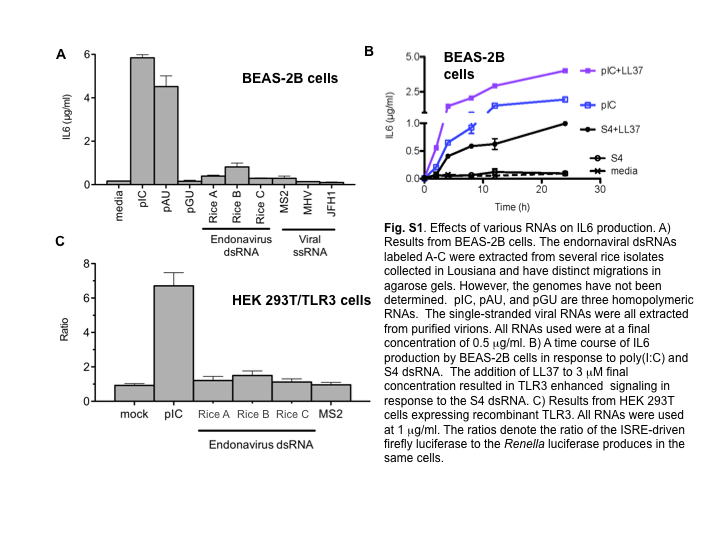

Supplement: Figure S1 — Effects of various RNAs on IL6 production. A) Results from BEAS-2B cells. The endornaviral dsRNAs labeled A–C were extracted from several rice isolates collected in Lousiana and have distinct migrations in agarose gels. However, the genomes have not been determined. pIC, pAU, and pGU are three homopolymeric RNAs. The single-stranded viral RNAs were all extracted from purified virions. All RNAs used were at a final concentration of 0.5 µg/ml. B) A time course of IL6 production by BEAS-2B cells in response to poly(I:C) and S4 dsRNA. The addition of LL37 to 3 µM final concentration resulted in TLR3 enhancing signaling in response to the S4 dsRNA or poly(I:C). C) Results from HEK 293T cells expressing recombinant TLR3. All RNAs were used at 1 µg/ml. The ratios denote the ratio of the ISRE-driven firefly luciferase to the Renilla luciferase produces in the same cells. (TIF) [file pone.0025837.s001.tif]

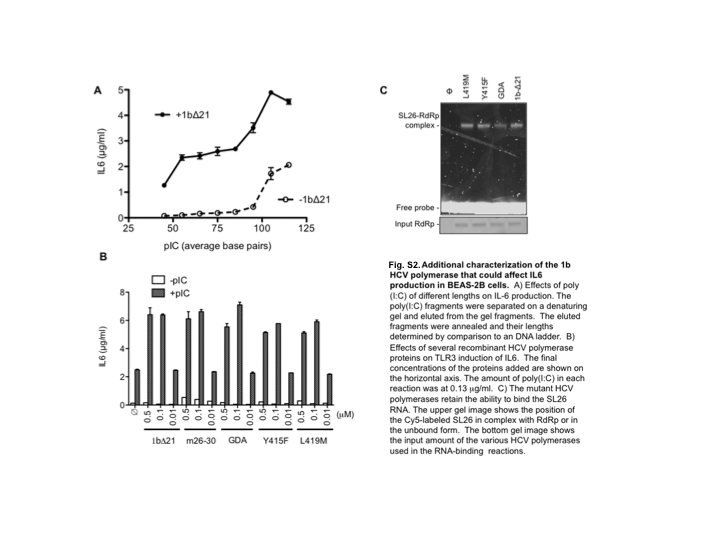

Supplement: Figure S2 — Additional characterization of the 1b HCV polymerase that could affect IL6 production in BEAS-2B cells. A) Effects of poly(I:C) of different lengths on IL-6 production. The poly(I:C) fragments were separated on a denaturing gel and eluted from the gel fragments. The eluted fragments were annealed and their lengths determined by comparison to an DNA ladder. B) Effects of several recombinant HCV polymerase proteins on TLR3 induction of IL6. The final concentrations of the proteins added are shown on the horizontal axis. The amount of poly(I:C) in each reaction was at 0.13 µg/ml. C) A demonstration that the mutant HCV polymerases retain the ability to bind the SL26 RNA. (TIF) [file pone.0025837.s002.tif]

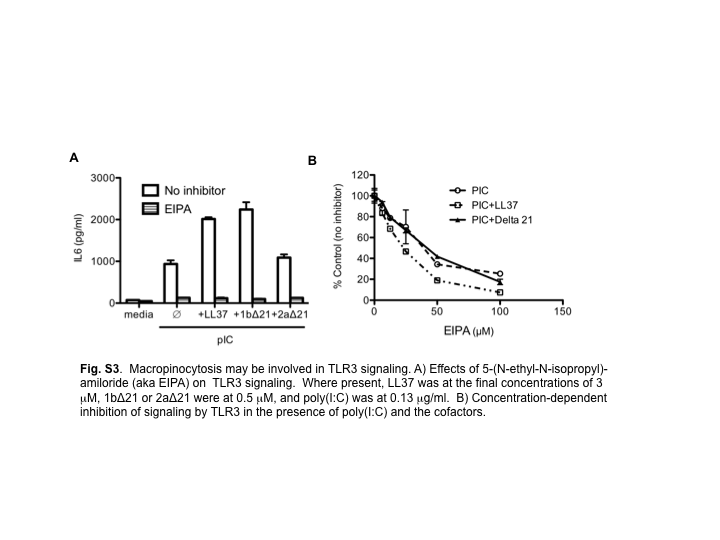

Supplement: Figure S3 — Macropinocytosis may be involved in TLR3 signaling. A) Effects of 5-(N-ethyl-N-isopropyl)-amiloride (aka EIPA) on TLR3 signaling. Where present, LL37 was at the final concentrations of 3 µM, 1bΔ21 or 2aΔ21 were at 0.5 µM, and poly(I:C) was at 0.13 µg/ml. B) Concentration-dependent inhibition of signaling by TLR3 in the presence of poly(I:C) and the cofactors. (TIF) [file pone.0025837.s003.tif]

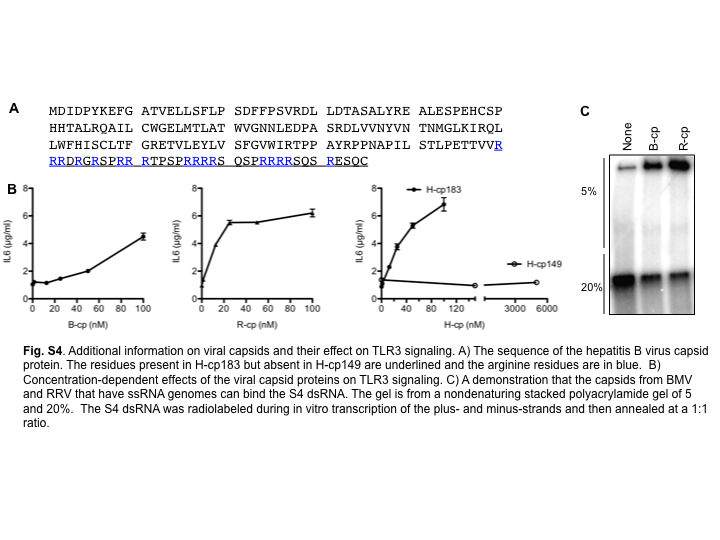

Supplement: Figure S4 — Additional information on viral capsids and their effect on TLR3 signaling. A) The sequence of the hepatitis B virus capsid protein. The residues present in H-cp183 but absent in H-cp149 are underlined and the arginine residues are in blue. B) Concentration-dependent effects of the viral capsid proteins on TLR3 signaling. C) A demonstration that the capsids from BMV and RRV that have ssRNA genomes can bind the S4 dsRNA. The gel is from a nondenaturing stacked polyacrylamide gel of 5 and 20%. The S4 dsRNA was radiolabeled during in vitro transcription of the plus- and minus-strands and then annealed at a 1∶1 ratio. (TIF) [file pone.0025837.s004.tif]

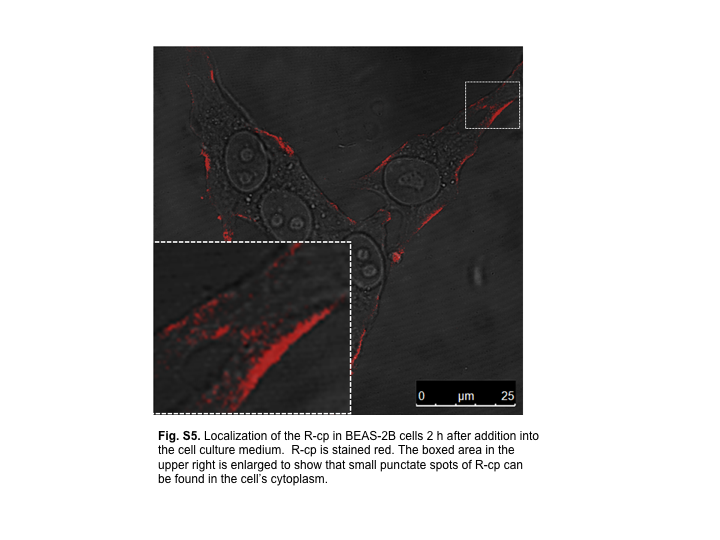

Supplement: Figure S5 — Localization of the R-cp in BEAS-2B cells 2 h after addition into the cell culture medium. R-cp is stained red. The boxed area in the upper right is enlarged to show that small punctate spots of R-cp can be found in the cell's cytoplasm. (TIF) [file pone.0025837.s005.tif]

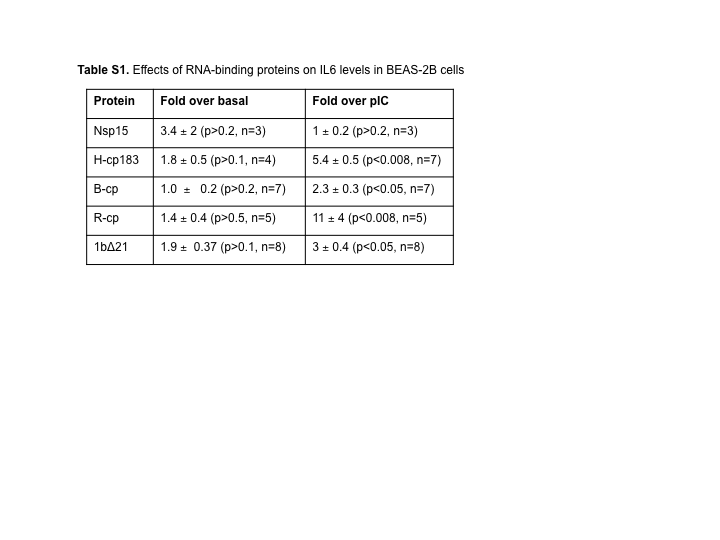

Supplement: Table S1 — Effects of RNA-binding proteins on IL6 levels in BEAS-2B cells. (TIF) [file pone.0025837.s006.tif]

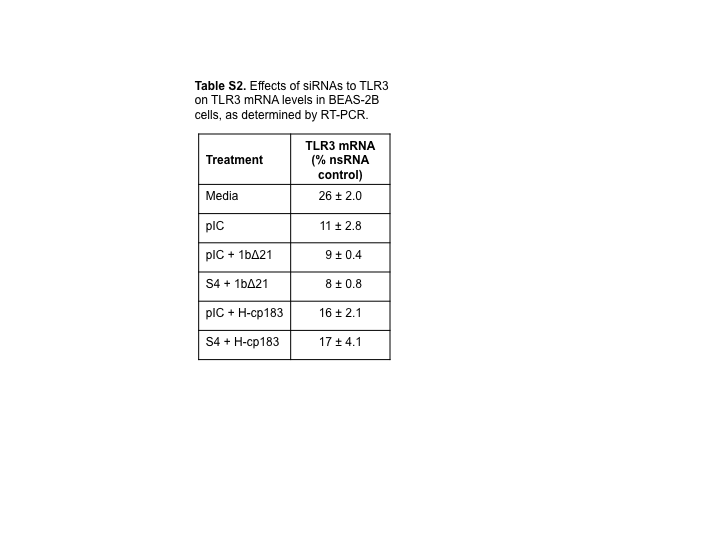

Supplement: Table S2 — Effects of siRNAs to TLR3 on TLR3 mRNA levels in BEAS-2B cells, as determined by RT-PCR. (TIF) [file pone.0025837.s007.tif]
